# The feasibility of assessing swallowing physiology following prolonged intubation after cardiovascular surgery

**DOI:** 10.1186/s40814-017-0199-7

**Published:** 2017-11-21

**Authors:** Stacey A. Skoretz, Terrence M. Yau, John T. Granton, Rosemary Martino

**Affiliations:** 1 School of Audiology and Speech Sciences, University of British Columbia, #421-2177 Wesbrook Mall, Vancouver, BC V6T 1Z3 Canada; 2 Department of Critical Care, University of Alberta, 2-124 Clinical Sciences Building, Edmonton, AB T6G 2B7 Canada; 3 Division of Cardiovascular Surgery, University Health Network, Toronto General Hospital, 200 Elizabeth Street, Toronto, ON M5G 2C4 Canada; 4 Division of Critical Care, Toronto General Hospital, 200 Elizabeth Street, Toronto, ON M5G 2C4 Canada; 5 Division of Respirology, Toronto General Hospital, 200 Elizabeth Street, Toronto, ON M5G 2C4 Canada; 6 Department of Speech-Language Pathology, University of Toronto, 160-500 University Ave, Toronto, ON M5G 1V7 Canada; 7 Division of Health Care and Outcomes Research, Krembil Research Institute, University Health Network, 399 Bathurst Street, Main Pavilion 11-331, Toronto, ON M5T 2S8 Canada

**Keywords:** Swallowing, Dysphagia, Intubation, Cardiovascular, Feasibility, Speech-language pathology, Videofluoroscopy

## Abstract

**Background:**

Dysphagia following prolonged intubation after cardiovascular (CV) surgery is common occurring in 67% of patients; however, this population’s swallowing physiology has never been prospectively evaluated using standardized methods. Hence, prior to conducting a larger study, our primary objective was to determine the feasibility of assessing swallowing physiology using instrumentation and validated interpretation methods in cardiovascular surgical patients following prolonged intubation.

**Method:**

From July to October 2011, we approached adults undergoing CV surgery at our institution who were intubated > 48 h. Those with a tracheostomy were excluded. Videofluoroscopic swallowing study (VFS) and nasendoscopy were completed within 48 h after extubation. Feasibility measurements included recruitment rate, patient participation, task completion durations, and the inter-rater reliability of VFS measures using the intraclass correlation coefficient (ICC). VFSs were interpreted using perceptual rating tools (Modified Barium Swallow Measurement Tool for Swallow Impairment™^©^ and Penetration Aspiration Scale) and objective displacement measurements (hyoid displacement and pharyngeal constriction ratio).

**Results:**

Of the 39 patients intubated > 48 h, 16 met inclusion criteria with three enrolled and completing the VFS. All refused nasendoscopy. Across all VFSs, rating completion time ranged from 14.6 to 51.7 min per patient with ICCs for VFS scales ranging from 0.25 (95% CI − 0.10 to 0.59) to 0.99 (95% CI 0.98 to 0.99).

**Conclusions:**

This study design was not feasible as recruitment was slow, few patients participated, and no patient agreed to all procedures. We discuss necessary methodological changes and lessons learned that would generalize to future research.

## Background

Dysphagia occurs in two thirds of all patients who are intubated for 48 h or more following cardiovascular (CV) surgery [1, 2]. Not only does post-extubation dysphagia increase hospitalization costs [3]; patients with the disorder are at greater risk of pneumonia, reintubation, and death [4].

What is known about dysphagia frequency and swallowing physiology following prolonged intubation after CV surgery is limited by study design and the quality of the available literature [5]. Dysphagia incidence, as reported to date, is variable given these limitations. Available literature includes retrospective studies that rely on chart audit data and heterogeneous assessment methods [5]. Furthermore, many studies have inherent bias risk as they lack (1) assessor blinding to clinical data and/or outcomes and (2) validated rating methods for instrumental assessments [5]. To date, no study has analyzed swallowing physiology using standardized ratings in consecutively enrolled patients following prolonged intubation after CV surgery. Hence, the feasibility of a rigorous research paradigm that includes CV surgical patients from intensive care as well as blinded raters using validated tools has yet to be determined. Our primary objective was to determine the feasibility of using validated and objective interpretation measures for videofluoroscopy in conjunction with nasendoscopy to assess swallowing and upper airway physiology on prospectively enrolled CV surgery patients following prolonged intubation. Our secondary objective was to explore the tolerability and impact of this study on patients and nursing practice. These findings will be used to inform a future large-scale study to systematically evaluate the incidence of dysphagia and comprehensively assess the swallowing physiology of this patient population.

## Methods

### Participants

From July 1 to October 31 2011, patients at our institution intubated for > 48 h following CV surgery were approached for study participation and consented by an independent research assistant. Eligible patients included those > 18 years of age who were extubated within the previous 48 h. We excluded patients with a history of dysphagia, tracheotomy, head/neck cancer, or neurological disorders including stroke or seizures. Also excluded were patients deemed inappropriate for study participation by the attending medical team, including those with reduced consciousness, medical instability or who were nil per os (NPO) due to gastroenterologic complications. Our institutional research ethics board approved this study, and all participants or surrogate decision-makers provided written informed consent prior to participation.

### Study process

Within 48 h following extubation, the patient’s swallow was assessed using a videofluoroscopic swallowing study (VFS) conducted by a speech-language pathologist blinded to all clinical data. In addition, these patients were approached to undergo flexible nasendoscopy by otolaryngology to assess for upper airway pathology. At the completion of instrumental testing, we invited the patient and attending nurse to provide anonymous feedback on the study process using a self-administered impact questionnaire. We measured process and resource feasibility including recruitment rate (aim of ≥ two patients/week in order to meet a convenience sample size of *N* = 30), number of eligible patients, losses to enrollment, patient participation with all instrumental protocols, task completion times, and the inter-rater reliability of the VFS measures (aim of ICC ≥ 0.60).

### Videofluoroscopic swallow study

#### Imaging

Fluoroscopy was conducted using a Toshiba Ultimax System MDX-8000A (Toshiba America Medical Systems Inc., Tustin, CA) in the lateral position. Using continuous pulse, the image was captured in uncompressed form using a TIMS 2000 DICOM system (Forest Imaging, Chelmsford, MA) at a rate of 30 frames per second. Image collimation allowed for views of the anterior lip margins, superior aspect of the nasal passages, posterior margins of the cervical vertebrae, and cervical esophagus. A visible scalar with known dimensions (a quarter) was placed submentally in the image field for calibration of image magnification.

#### Videofluoroscopic swallow study procedure

Each patient was presented with various fluid and food textures combined with E-Z-EM barium contrast agents (E-Z-EM Inc., Lake Success, NY) according to institutional standard practice and preparation. The trials were presented as follows: (1) thin liquid with diluted liquid Polibar Plus barium sulfate suspension (47% *w*/*v*; 3 × 5 ml boluses by spoon, 2 × 15 ml boluses by cup), (2) applesauce mixed with powdered barium (28% *w*/*w*; 3 × 5 ml boluses by spoon), (3) ½ Peak Frean digestive cookie coated with barium paste (60% *w*/*w*), and (4) sequential cup sips of the thin liquid barium dilution (47% *w*/*v*; 100-ml maximum). If the patient exhibited aspiration of any texture, a larger bolus of that texture was not administered. The testing sequence was discontinued in its entirety if aspiration of applesauce occurred. For all trials, except for sequential cup sipping, a cued-swallow paradigm was used with instruction to hold the bolus in the oral cavity and then swallow on command.

##### Videofluoroscopic swallow study measures

Following VFS completion, we conducted two types of evaluations using (i) standardized VFS rating tools and (ii) objective displacement measurements.

i. *Standardized VFS rating tools.* Each VFS was scored in its entirety using two standardized, validated, and reliable tools: the Modified Barium Swallow Impairment Profile (MBSImp™^©^; Northern Speech Services, Gaylord, MI) [6] and the Penetration Aspiration Scale (PAS) [7]. These tools are complementary, measuring swallowing impairment and airway protection respectively.

One MBSImp™^©^ certified rater (SAS) completed the MBSImp™^©^ for each bolus administration while blinded to clinical data. The MBSImp™^©^ is a standardized tool used to quantify severity of oral and pharyngeal impairment through assessment of 17 physiologic components. Each component has a rank-ordered scoring system, ranging from a three- to a five-point scale, with increasing scores indicating greater impairment. A priori, we excluded two components, pharyngeal constriction and esophageal clearance, the former, as it requires an anterior posterior fluoroscopic view, which cannot be obtained from this patient population given their restricted mobility at the time of testing and, the latter, as it was beyond the scope of this study. For each patient, each bolus administration was scored individually according to published guidelines [6], with the patient receiving the highest, most impaired score if a texture or bolus volume was not administered due to safety concerns.

Two independent raters (SAS and RM) were blinded to each other and all clinical data conducted PAS scoring for each bolus administration. The PAS [7] is an eight-point ordinal severity scale scoring the depth of airway invasion by the bolus, whether it is expelled from the airway as well as any patient reaction. It ranges from one (material does not enter the airway) to eight (material enters the airway, passes below the vocal folds, and no effort is made to eject).

ii. *Displacement measurements*. Two independent raters (SAS and RM) blinded to each other and all clinical data, conducted frame selection and displacement measurements for each 5 and 15-ml bolus using ImageJ (National Institutes of Health, Bethesda, MD) focusing on two domains: hyolaryngeal excursion and pharyngeal constriction [8–10]. These specific features are associated with successful propulsion of food or fluid through the pharynx into the esophagus thereby preventing laryngeal penetration, tracheal aspiration, and/or pharyngeal residue [9–15].

Hyoid excursion during each swallow was calculated according to two techniques: (1) as an absolute trajectory measurement in millimeters (mm) quantifying the anterior-superior displacement of the hyoid bone from its rest position [10, 16] and (2) as an internally scaled calculation of separate anterior and superior hyoid movement using the patient’s cervical vertebrae as a reference [9, 17]. We utilized two fluoroscopy images per bolus for both techniques: one with the hyoid at rest and one at maximal anterior-superior displacement. The hyoid “rest” frame was defined differently for each technique: (1) during bolus hold prior to swallow initiation for the absolute measurements and (2) during the lowest hyoid position following swallow completion for the scaled measurements. Frame selection, anatomical tracing specifications, and displacement calculations for both techniques were completed according to previously published methods [9, 10, 13, 17].

We measured pharyngeal constriction during the swallow using ImageJ through a pharyngeal constriction ratio (PCR) calculation [10, 13, 16]. For this measurement, we defined the bolus hold frame (PA_HOLD_) as the hold during the 5-ml thin liquid bolus. The maximum pharyngeal contraction frames (PA_MAX_) were defined as the frame with maximal pharyngeal contraction during each of the following: 5- and 15-ml thin liquid and 5-ml pureed boluses. Following pharyngeal space tracing for each frame, we calculated the pharyngeal constriction ratio by dividing the pharyngeal area (cm^2^) of the selected frames (PA_MAX_/PA_HOLD_) [10, 13, 16].

### Study impact questionnaires and patient variables

Following VFS completion, the patient and attending nurse were asked to complete the study impact questionnaires (Table 1). The questionnaires utilized a 5-point Likert-like scale rating patient comfort, study impact on nursing workload and perceived study value while providing an open-ended section for comments. Once completed, we instructed the patient and/or nurse to deposit their questionnaire in a locked box placed on the hospital unit. Following unblinding, the first author (SAS) recorded patient variables such as demographics (age, gender) and operative information (surgery type, intubation duration). The first author (SAS) entered and analyzed all data following VFS rating completion and unblinding to clinical data.

**Table 1:** Study impact questionnaires

Question		Rating scale				
		1	2	3	4	5
Patient questionnaire	How did you find the x-ray swallow test?	Easy	Somewhat easy	Adequate	Somewhat difficult	Very difficult
	How did you find the nasendoscopy?					
	Overall, how was your experience with this research?					
	How was this form to complete?					
Nursing questionnaire	In your opinion, to what degree did this study affect the delivery of patient care?	Not at all	Very little	A little	Somewhat	A lot
	How did the participation of this study fit into your daily tasks?	Easily	Somewhat easily	Adequately	With difficulty	With great difficulty
	How were you able to accommodate being part of the videofluoroscopic swallow study?					
	How was this form to complete?	Easy	Somewhat easy	Adequate	Somewhat difficult	Very difficult
Comments:						

### Scoring and statistical analyses

For each patient, we scored each bolus administration individually with both the MBSImp™^©^ and PAS. We derived overall impression impairment scores (OI) for each of the MBSImp™^©^ components for each patient according to published standards, namely (1) levying the maximum score should a bolus texture or volume not be administered due to safety concerns and (2) using the patient’s worst score for each component regardless of texture or volume [6]. We derived the total oral impairment (components 1–6) and total pharyngeal impairment (components 7–12 and 14–16) scores for each patient by the summation of each OI score from the appropriate oral and pharyngeal components. We dichotomized PAS scores as either normal (PAS scores 1 or 2) or abnormal (PAS scores 3 to 8) airway protection for each bolus administration [18, 19] and summarized these data as frequency counts.

We reported the approximate duration in minutes for patient transport to medical imaging, patient VFS participation, and task completion (VFS preparation, study archiving). Completion time for (1) frame selection and deriving displacement measurements, (2) MBSImp™^©^, and (3) PAS ratings were reported in minutes as means with standard deviations (SD). We summarized questionnaire responses descriptively and according to frequency counts. Across each bolus administration as described above, we explored the single measures inter-observer agreement using absolute agreement two-way mixed intraclass correlation coefficient (ICC) with 95% confidence intervals (CI) for (1) the PAS (i.e., classification agreement across the eight-point scale), (2) frame selection (i.e., selection agreement for the following frames: rest/hold, maximum hyoid displacement, and maximum pharyngeal constriction), and (3) displacement measurements (i.e., hyoid trajectory, scaled hyoid displacement, and pharyngeal constriction ratio). These ICC estimates were crossed two-way analyses of variances adjusting for repeated measures. Statistical analyses were conducted using IBM SPSS version 22 (IBM Corporation, Armonk, NY). Radar plots were created using OriginPro 9.1 (OriginLab Corporation, Northampton, MA).

## Results

### Patient recruitment and characteristics

**Fig. 1:**
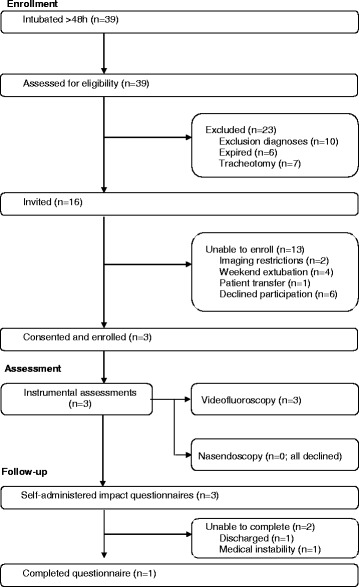
Study enrollment

During the 4-month study period, 39 patients required intubation for more than 48 h (Fig. 1). Of those, 16 met the inclusion criteria with three patients agreeing to participate for an approximate recruitment rate of one patient every 5 weeks. Of the remaining 13 patients, six declined participation, six were not approached due to institutional and operational restrictions (i.e., no weekend coverage and diagnostic imaging suite downtime), and one patient was transferred off-service.

Our study consisted of two males and one female with ages ranging from 37 to 71 years. Intubation durations for patient 1 (P1), patient 2 (P2), and patient 3 (P3) were 53.3, 49.3, and 82.5 h respectively. Other demographic and medical information was collected but not included here to preserve patient anonymity in light of the small sample. At the time of study participation, all patients were in intensive care requiring 24-h one-to-one nursing care. All enrolled patients refused nasendoscopy.

### Videofluoroscopic swallow study

The videofluoroscopic swallow study (VFS) was completed 30, 25, and 37 h following extubation on P1, P2, and P3 respectively. All consistencies and volumes were administered to P1 and P2. For P3, the VFS protocol was discontinued following two of the three pureed (5 ml) boluses due to safety concerns. All patients were NPO prior to the VFS. Artifacts on the radiographic image included a central line (P1 and P2) and an in situ nasogastric feeding tube (P3).

For each VFS, patient preparation and transport time to and from the fluoroscopy suite ranged from 30 to 60 min. Prior to patient arrival, room and equipment preparation required an average of 10 min. While in the imaging suite, patient setup and the VFS were completed within 15 min. Following the completion of each VFS, data transfer and study archiving took approximately 1 h. A total of three study personnel were involved in the VFS directly: an x-ray technician, a speech-language pathologist (SAS), and a research assistant. The patient’s nurse and clinical speech-language pathologist were also in attendance.

### Videofluoroscopic measures

Videofluoroscopic measurements were completed for all three patients. The time to complete frame selection and measurement for each method ranged from less than 2 to 5.9 min per bolus administration. The total time to complete each measurement across all targeted bolus volumes ranged from 14.6 to 51.7 min for each VFS. For the displacement measures, the frame selection ratio ICC ranged from 0.98 (0.96 to 0.99) to 0.99 (0.98 to 0.99). Contrastively, the displacement measurement ICC ranged from 0.25 (− 0.10 to 0.59) to 0.90 (0.76 to 0.96). The ICC for the PAS was 0.92 (0.83 to 0.96). Each VFS measure and its corresponding task completion time and inter-rater reliability (as applicable) is summarized in Table 2.

**Table 2:** Task completion time and inter-rater reliability according to VFS measure

VFS measure	Completion time^a^		ICC (95% CI)	
	Per bolus administration	Per VFS	Frame selection ratio	Measurement
MBSImp™^©^	5.9 (0.8)	51.7 (16.5)	N/A	N/A
PAS	1.9 (0.9)	17.3 (10.1)	N/A	0.92 (0.83 to 0.96)
Absolute hyoid displacement^b^	5.2 (2.5)	38.7 (21.5)	0.99 (0.98 to 0.99)	0.90 (0.76 to 0.96)
Scaled hyoid displacement^b^	1.9 (0.5)	14.6 (4.8)	0.98 (0.96 to 0.99)	0.26 (− 0.05 to 0.52)
Pharyngeal constriction^b^	4.5 (1.0)	33.3 (10.1)	0.99 (0.98 to 0.99)	0.25 (− 0.10 to 0.59)

*Note*. *VFS* videofluoroscopic swallow study, *CI* confidence interval, *MBSImp™* ^*©*^ Modified Barium Swallow Impairment Profile, *N/A* not applicable, *PAS* Penetration Aspiration Scale

^a^Reported in minutes; mean (SD)

^b^Displacement measurements conducted on 5 and 15-ml bolus volumes only

#### Standardized VFS rating tools

**Fig. 2:**
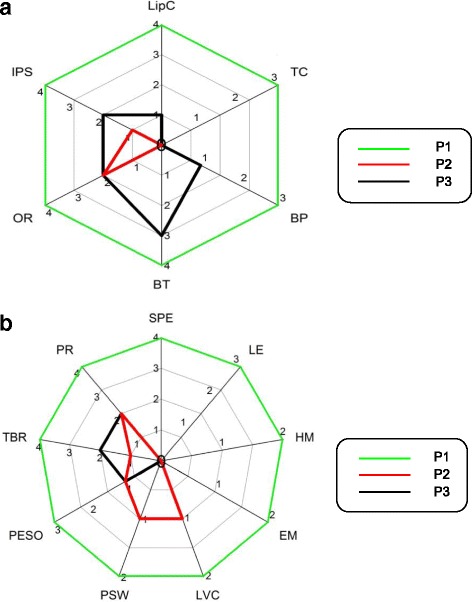
**a** MBSImp™^©^ oral components across patients. *Note*. LipC = lip closure, TC = tongue control, BP = bolus preparation, BT = bolus transport, OR = oral residue, IPS = initiation of pharyngeal swallow; scoring, 0 = normal, ≥ 1 = impairment. **b** MBSImp™^©^ pharyngeal components across patients. *Note*. SPE = soft palate elevation, LE = laryngeal elevation, HM = hyoid movement, EM = epiglottic movement, LVC = laryngeal vestibule closure, PSW = pharyngeal stripping wave, PESO = pharyngoesophageal segment opening; TBR = tongue base retraction; PR = pharyngeal residue; scoring: 0 = normal, ≥ 1 = impairment

P3 received the maximum overall impression (OI) score across all individual MBSImp™^©^ oral and pharyngeal components (Fig. 2a, b). As a result, P3 received the highest total oral and total adjusted pharyngeal scores of 22 and 26 respectively, exhibiting the most severe swallowing impairment of all patients in our study. Of the two remaining patients, P1 received a total oral impairment score of nine as compared to three for P2, with greater impairment on lip closure, bolus preparation, bolus transport, and the initiation of the pharyngeal swallow. Conversely, P2 received a total pharyngeal impairment score of six as compared to five for P1, with greater impairment on laryngeal vestibule closure and pharyngeal stripping wave components. Of the remaining pharyngeal components, P1 and P2 differed only on tongue base retraction with OI scores of two and one respectively. Of the oral components, all three patients exhibited impairment on oral residue and initiation of the pharyngeal swallow. Additionally, all patients exhibited impairment on the following pharyngeal components: pharyngoesophageal segment opening, tongue base retraction, and pharyngeal residue. Across all bolus administrations, airway protection of P1 and P2 was rated as normal (PAS scores ≤ 2) whereas the airway protection of P3 was rated abnormal (PAS scores ≥ 3). Clinically, P1 and P2 began regular texture diets following VFS completion while P3 remained NPO with enteral feeding via nasogastric feeding tube.

#### Displacement measurements

Hyoid displacements are presented in Table 3. In P3, we found the smallest median (interquartile range (IQR)) absolute hyoid displacement [10, 16]: 8.3 mm (2.3) and 8.3 mm (1.7) with thin (5 ml) and pureed (5 ml) textures respectively. Contrastively, in P1, we found the largest median (IQR) displacements 12.7 mm (1.7) and 14.8 mm (2.1) respectively. Absolute displacement median values increased with both increasing volume as well as increasing texture viscosity for P1 and P2 whereas median values for P3 remained consistent regardless of stimulus change. Scaled anterior and superior hyoid displacement measurements (%C2–4 distance) [9, 17] were similarly patterned with the (1) smallest median values regardless of texture for P3 and (2) largest median values for P1 with thin fluid boluses.

**Table 3:** Hyoid displacement measurements according to patient

Case	Absolute hyoid displacement (mm)			Scaled hyoid displacement (%C2–4 distance)					
				Anterior hyoid displacement			Superior hyoid displacement		
	Thin liquid		Pureed	Thin liquid		Pureed	Thin liquid		Pureed
	5 ml	15 ml	5 ml	5 ml	15 ml	5 ml	5 ml	15 ml	5 ml
P1	12.7 (1.7)	14.2 (3.2)	14.8 (2.1)	41.6 (18.7)	52.4 (6.0)	40.8 (30.7)	18.5 (25.8)	31.0 (10.9)	36.9 (25.9)
P2	9.8 (1.8)	9.8 (1.6)	9.9 (2.1)	23.1 (41.0)	27.4 (8.1)	24.3 (9.4)	27.9 (57.8)	33.6 (17.0)	36.7 (30.8)
P3	8.3 (2.3)	NT	8.3 (1.7)	17.9 (12.2)	NT	22.5 (5.8)	13.9 (14.5)	NT	17.8 (9.5)

*Note*. Values are reported as median (IQR)

*IQR* interquartile range, *NT* not tested

**Fig. 3:**
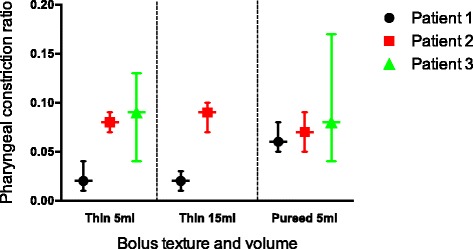
Pharyngeal constriction ratio by patient according to volume

When comparing PCR measurements across patients according to bolus texture and volume (Fig. 3), we measured the smallest median (IQR) pharyngeal constriction ratio with thin fluid boluses (5 ml) for P1 (0.02 [0.03]) and the largest with P3 (0.09 [0.09]) for the same bolus texture and volume. Within patients across bolus texture and volumes, PCR values decreased with increasing viscosity for P2 and P3 with the inverse measured for P1.

### Study impact questionnaires

#### Patient comfort questionnaires

One patient completed the questionnaire. Ratings were “adequate” for VFS and overall study participation and “easy” for questionnaire completion. This patient would have preferred more information during the consent process about the instrumental assessment. Of the two remaining patients, one was medically incapable of completing the questionnaire and one was discharged prior to completion.

#### Workload impact questionnaires

All attending nurses, three cardiovascular intensive care unit (CVICU) nurses and one nursing student, completed the questionnaire. All nurses reported the study fit well with the daily tasks and the three felt VFS accommodation was “easy” or “adequate”. Half of the nurses reported the study’s impact on patient care delivery was minimal. Nursing suggestions for study modifications included (1) further advance notice regarding the procedure timing in order to allow sufficient lead time to ready the patient for transport and (2) frequent confirmation with the nursing staff regarding the patient’s hemodynamic stability and inotropic support requirements prior to scheduling transport.

## Discussion

Evidenced by a slow recruitment rate with no patient agreeing to all procedures, our study as designed was not feasible. While we are the first to conduct a study of this nature with this patient population, the low enrollment and missing data were devastating. This experience has afforded us the opportunity to revisit our methodology prior to conducting our larger study. Despite these failures, we were able to minimize bias risk through instrumental assessment on all enrollees, swallow study interpretation using validated rating scales, and assessor blinding throughout the study. Our findings demonstrate that for the success of future large-scale studies, we will maintain select aspects of our design in an effort to reduce unnecessary bias; however, changes need be considered to enhance recruitment and consent. For example, changes to the enrollment criteria, diagnostic methods, and interpretation measures may meet these goals.

Although we targeted the patient group at the highest risk of developing dysphagia [1, 2], our recruitment rate was slow: a rate of less than one patient per month. At our current rate, meeting a hypothetical sample size of 100 would take 10 years. We suggest (1) revising our definition of prolonged intubation to include those intubated for 24 h or longer, (2) increasing research staff availability over weekends, (3) increasing the post-extubation instrumental assessment window from 48 to 72 h, and (4) expanding the study to include multiple centers. These changes must be considered carefully as they may (1) increase the study cost, (2) increase burden on the institution and staff, and (3) require larger sample sizes for particular outcome comparisons. For example, increasing the post-extubation instrumental assessment window could be problematic for a study also targeting dysphagia incidence and/or swallow outcome comparisons given that swallowing physiology changes rapidly following extubation. It has been reported that swallow timing may improve within 48 h following extubation [20] with aspiration frequency decreasing within 8 h [21]. As a result, a broader range in post-extubation assessment times would require patient stratification thereby necessitating a larger sample size.

We incorporated aspects into our study that would provide information regarding study acceptance and consent. Our low enrollment limited our ability to make inferences regarding study impact on either nursing or patients; however, the responses can provide insight on how to improve study process and potentially increase our consent rate. All four nursing staff responded to our impact questionnaires and rated our protocol favorably. In contrast, only one of the three patients completed their questionnaires. Two patients did not respond due to discharge and medical instability respectively. Our single patient respondent suggested that in order to improve the consenting process, the instrumental assessment should be explained more fully. Given the limited enrollment and all patients refusing nasendoscopy, the study protocol should be altered to include only one instrumental procedure. For future studies, we will review the consenting process not limited to the information presented to the participants. Nursing commented on timing issues regarding transport to radiology. We hypothesize that a bedside instrumental swallowing assessment may be favored by both nursing and patient. It would eliminate patient transport outside of the ICU and in so doing reduce (1) the number of staff required to conduct the assessment, (2) the time spent transporting the patient, and (3) reduce patient burden. Moving forward, we recommend the conduct of structured interviews with potential study participants including patients, caregivers, and nursing staff. This would enable investigators to have a greater understanding of the procedures with which they are willing to participate, when they would be willing to participate and their preferences for the consenting process.

This is the first study to provide a detailed assessment of swallowing physiology in this population using a valid and reliable interpretation method of videofluoroscopy. All three patients exhibited swallowing impairment across multiple areas. However, while the swallowing impairments found in our study were similar to those reported previously [22, 23], we did note an incongruence between the MBSImp™^©^ scores for P1 and P2 with what was observed clinically. Both patients were able to consume regular texture diets without difficulty. As a result, in conjunction with valid and objective interpretation methods for videofluoroscopy, we recommend that future research also incorporate a means by which to determine clinically relevant dysphagia.

To the best of our knowledge, we are the first to report on swallowing kinematics on recently extubated patients following CV surgery. When comparing inter-rater agreement across all displacement measures, caution needs to be taken as these estimates are based on only three patients. The reliability for frame selection across all measures and absolute hyoid displacement measurement was excellent [24]. In contrast, the reliability was poor for both scaled hyoid displacement measurement as well as pharyngeal constriction ratio. This may be due to our very small sample size or patient factors. Due to the anatomical location of a radiographic artifact either along the cervical spine (i.e., central line) or in the pharynx (i.e., nasogastric tube) across each patient, it may be that these measures were most susceptible thereby accounting for the high variability between raters. During our reliability training, our sample studies did not include these artifacts. For this population specifically, displacement and pharyngeal constriction are important measures due to the relative inactivity of the laryngeal and pharyngeal musculature during intubation and the potential effect of the endotracheal tube on swallow function [25, 26]. Addressing measurement reliability prior to enrollment using fluoroscopy samples with similar radiographic artifacts would not only be prudent but critical.

This feasibility study, which by definition is descriptive and exploratory in nature, has several additional limitations. Not only was this study conducted for a very limited duration, we were unable to approach all potential participants for study participation. This limited our enrollment resulting in an inability to report dysphagia frequency. In addition, the small sample size precluded statistical comparisons of our VFS measures, with the reported swallowing characteristics unlikely to be representative of this population as whole. Our patients’ acuity restricted our VFS assessments as all patients presented with either a central line or nasogastric feeding tube.

## Conclusions

In conclusion, while we were able to conduct VFSs successfully on our participants shortly after extubation following CV surgery, our inability to successfully execute our complete study protocol and slow enrollment rate rendered our design not feasible. We have, however, learned lessons throughout the process which inform the changes necessary for future feasibility studies and protocols. Prior to the conduct of future large prospective studies, investigators should engage both patients and staff to gain a greater understanding of the procedures with which they are willing to participate and to improve the consenting process. Other design changes may include patients intubated for 24 h or more while allowing for instrumental swallowing assessments throughout the entire seven-day week. Also, patient burden can be minimized with only one instrumental procedure and depending on study objectives, a bedside instrumental protocol such as fiberoptic endoscopic evaluation of swallowing (FEES) may be easier to implement for acute patient populations. However, due to the limited knowledge surrounding this population’s swallowing physiology, some future studies should still incorporate VFS. This instrumentation permits kinematic, temporal, and event sequence measurement. In order to maintain objective interpretation, it is crucial to use standardized rating tools and inter-rater reliability calibrated a priori to ensure consistency in displacement measurements. Although dysphagia incidence and swallowing physiology of this population still remains elusive, our study provides the necessary foundation for future investigations focused on dysphagia frequency and swallow characteristics in post-extubation patients.

## Abbreviations

BP: Bolus preparation
BT: Bolus transport
CA: California
CI: Confidence interval
CV: Cardiovascular
CVICU: Cardiovascular intensive care unit
EM: Epiglottic movement
FEES: Fiberoptic endoscopic evaluation of swallowing
HM: Hyoid movement
ICC: Intraclass correlation coefficient
ICU: Intensive care unit
IPS: Initiation of pharyngeal swallow
IQR: Interquartile range
LE: Laryngeal elevation
LipC: Lip closure
LVC: Laryngeal vestibule closure
MA: Massachusetts
MBSImp™^©^: Modified Barium Swallow Measurement Tool for Swallow Impairment
MD: Maryland
MI: Michigan
N/A: Not applicable
NPO: Nil per os
NT: Not tested
NY: New York
OI: Overall impression impairment scores
OR: Oral residue
P1: Patient 1
P2: Patient 2
P3: Patient 3
PAS: Penetration Aspiration Scale
PCR: Pharyngeal constriction ratio
PESO: Pharyngoesophageal segment opening
PR: Pharyngeal residue
PSW: Pharyngeal stripping wave
RM: Rosemary Martino
SAS: Stacey A. Skoretz
SD: Standard deviation
SPE: Soft palate elevation
TBR: Tongue base retraction
TC: Tongue control
VFS: Videofluoroscopic swallowing study
